# Synchronization through nonreciprocal connections in a hybrid hippocampus microcircuit

**DOI:** 10.3389/fncir.2013.00120

**Published:** 2013-07-23

**Authors:** Markus M. Hilscher, Katarina E. Leão, Richardson N. Leão

**Affiliations:** ^1^Neurodynamics Lab, Department of Neuroscience, Uppsala UniversityUppsala, Sweden; ^2^Brain Institute, Federal University of Rio Grande do NorteNatal, Brazil

**Keywords:** hippocampus, synchronization, dynamic clamp, *I*_*h*_, AMPA, NMDA

## Abstract

Synchronization among neurons is thought to arise from the interplay between excitation and inhibition; however, the connectivity rules that contribute to synchronization are still unknown. We studied these issues in hippocampal CA1 microcircuits using paired patch clamp recordings and real time computing. By virtually connecting a model interneuron with two pyramidal cells (PCs), we were able to test the importance of connectivity in synchronizing pyramidal cell activity. Our results show that a circuit with a nonreciprocal connection between pyramidal cells and no feedback from PCs to the virtual interneuron produced the greatest level of synchronization and mutual information between PC spiking activity. Moreover, we investigated the role of intrinsic membrane properties contributing to synchronization where the application of a specific ion channel blocker, ZD7288 dramatically impaired PC synchronization. Additionally, background synaptic activity, in particular arising from NMDA receptors, has a large impact on the synchrony observed in the aforementioned circuit. Our results give new insights to the basic connection paradigms of microcircuits that lead to coordination and the formation of assemblies.

## Introduction

Spike synchronization is crucial for motor and sensory functions; however, excessive coordination of neuronal firing is associated to pathological conditions (Paluszkiewicz et al., 2011). Indeed, synchronization of neuronal activity is a key concept within Hebb's theories of learning and memory (Hebb, 1949; Lopes-dos-Santos et al., 2011). Hebb proposed that organized activation of a neuronal group of neurons leads to the formation of neuronal assemblies, considerably increasing the efficiency of their inputs (Abeles et al., 1994). There is a large body of evidence supporting one of Hebb's original ideas that synchronization of neuronal firing is a direct result of connectivity (Perin et al., 2011) but intrinsic membrane properties can also contribute (or impede) the organization of assemblies (Varga et al., 2008).

In simple terms, two given cells or group of cells *A* and *B* can synchronize their firing if *A* and *B* are reciprocally connected or if a pacemaker cell, or group of cells, connects to both *A* and *B* (Hansel et al., 1995; Tort et al., 2007). Of note, both effects seem to take part in the generation of rhythmic patterns in the hippocampus; for example, while gamma oscillations are likely to arise form intrinsic network properties (Wang and Buzsáki, 1996), theta oscillations would depend both on intrinsic connectivity and pacemaker inputs from the medial septum (Colom, 2006).

Whilst it was first assumed that excitatory connections are the most important players in synchronization of neural activity, the role of inhibition in producing coordinated firing became evident about two decades ago (Van Vreeswijk et al., 1994). Synchronization through inhibition has been demonstrated *in vitro*, *in vivo* and in computer models (Van Vreeswijk et al., 1994; Cobb et al., 1995; Wang and Buzsáki, 1996). Vreeswijk and others showed that excitatory connections among neurons lead to synchronization in modeled neurons if synaptic events are short and have no delay (Van Vreeswijk et al., 1994). On the other hand, inhibitory synapses are more prone to produce coordination if the rise time of postsynaptic inhibitory currents are slower than the duration of the action potential (Van Vreeswijk et al., 1994). Apart from synaptic kinetics, voltage dependent ionic currents such as the hyperpolarization-activated cation current (*I*_*h*_) have also been mechanistically correlated to synchronization (Bal and McCormick, 1997). By regulating the membrane potential, *I*_*h*_ can make the neuron more or less susceptible to incoming excitation (Bal and McCormick, 1997), fire rebound action potentials after incoming inhibition (Leao et al., 2006a) or differentially balance excitatory and inhibitory inputs (Leão et al., 2011).

To date, most studies investigating the mechanisms behind synchronization and assembly formation have investigated either synaptic activity or membrane function on coordination of activity (Wang et al., 2012). Understanding the interplay between connectivity and membrane properties on the coordination of spiking activity has been lacking. Here, our study aims to investigate how different connection schemes in a hippocampal microcircuit influence the assurgency of synchronization. A pair of pyramidal cells (PCs) in hippocampal slices were virtually connected to each other and/or to a virtual interneuron using real-time computation environment (Leao et al., 2006a). We found that a circuit constituted by a nonreciprocal excitatory connection between PCs that in turn received inhibitory synapses from a virtual interneuron, showed the greatest synchronization. Subsequently, pharmacological agents were applied to the preparation to elucidate the role of PC *I*_*h*_ or uncoordinated synaptic activity in coordinating PC spikes in the aforementioned microcircuit.

## Materials and methods

### Electrophysiology

Horizontal hippocampal slices from C57B6 mice (postnatal day 12–25) were obtained as previously described (Leão et al., 2009). All experiments have been approved by the Swedish Animal Welfare authorities and follow Uppsala University guidelines for the care and usage of laboratory animals. In summary, brains were rapidly removed and placed in ice-cold sucrose/artificial cerebrospinal fluid (ACSF) consisting of the following (in mM): KCl, 2.49; NaH_2_PO_4_, 1.43; NaHCO_3_, 26; glucose, 10; sucrose, 252; CaCl_2_, 1; MgCl_2_, 4. Horizontal 400-μm-thick slices containing the hippocampus were cut using a vibratome (VT1200, Leica, Microsystems) and were subsequently moved to a submerged holding chamber containing normal ACSF (in mM: NaCl, 124; KCl, 3.5; NaH_2_PO_4_, 1.25; MgCl_2_, 1.5; CaCl_2_, 1.5; NaHCO_3_, 30; glucose, 10), constantly bubbled with 95% O_2_ and 5% CO_2_ and kept at 35°C for 1 h then maintained at room temperature. The slices were transferred to submerged chamber under an upright microscope equiped with DIC optics (Olympus) and perfused with at 30°C oxygenated ASCF (1–1.25 ml/min). Patch pipettes from borosilicate glass capillaries (GC150F-10 Harvard Apparatus) were pulled on a vertical puller (Narishige, Japan) with resistance around 5 MΩ. Pipettes were filled with internal solution containing (in mM): KCl, 17.5; K-gluconate, 122.5; NaCl, 9; MgCl_2_, 1; Mg-ATP, 3; GTP-Tris, 0.3; HEPES, 1; EGTA, 0.2 (pH was adjusted to 7.2 using KOH). Whole cell current clamp recordings were done using Dagan BVC 700 A amplifier (Dagan Corporation, Minneapolis, USA) and data was acquired using a 16-bit data acquisition card (National Instruments) and WinWCP and WinEDR softwares implemented by Dr. J. Dempster (University of Strathclyde, Glasgow, UK). A holding current was sometimes injected to either or both PCs to maintain a firing frequency above 2 Hz.

### Virtual connections and interneuron

A single compartment model of a fast-spiking interneuron was implemented as described in Wang and Buzsáki (1996). Changes in membrane potential were governed by sodium, potassium and leak currents, synaptic currents (*I*_syn_) and injected current (*I*_app_)

$$\begin{array}{l} C\frac{dV}{dt}=-g_{\text{Na}}m_{\infty }{\left(V\right)}^{3}h(V-E_{\text{Na}})-g_{k}n^{4}(v-E_{k})\\ \;-g_{L}(V-E_{L})-I_{\text{syn}}+I_{\text{app}} \end{array}$$

with

$$\begin{array}{l} m_{\infty }=\frac{\alpha_{m}}{\alpha_{m}+\beta_{m}}\\ \frac{dh}{dt}=(\alpha_{h}(1-h)-\beta_{h}(h))\varphi \\ \frac{dn}{dt}=(\alpha_{n}\left(1-n\right)-\beta_{n}(n))\varphi \end{array}$$

*C*, *V*, *E*, *t*, g and I denote capacitance density (μ*F* = cm^2^), voltage (mV), reversal potential (mV), time (ms), conductance density (mS/cm^2^) and current density (μA/cm^2^), respectively (*C* = 1, *g*_Na_ = 35, *g*_K_ = 9, *g*_L_ = 0.1, *E*_Na_ = 55, *E*_K_ = −90, *E*_L_ = −65, ϕ = 3.33). The rate functions α_*x*_ and β_*x*_ for *x* = *m*, *h* and n were defined as follows.

$$\begin{array}{l} \alpha_{m}=\frac{-0.1(V+35)}{\exp(-0.1(V+35))-1}\\ \beta_{m}=4\exp(-(V+60)/18)\\ \alpha_{h}=0.07\exp(-(V+58)/20)\\ \beta_{h}=\frac{1}{\exp(-0.1(V+28))+1}\\ \alpha_{n}=\frac{-0.01(V+34)}{\exp(-0.1(V+34))-1}\\ \beta_{n}=0.125\exp(-(V+44)/80) \end{array}$$

Both the pre- and postsynaptic currents (*I*_syn_) of the interneuron were calculated during the simulation. IPSCs and EPSCs were triggered when the membrane voltage of the presynaptic cell exceeded −20 mV. Pseudorandom inhibitory synaptic conductances were obtained by fitting a custom distribution on the distribution shown in Figure 1 of Maccaferri et al. (2000) and for excitatory currents we used the distribution of EPSC amplitudes of Figure 2 of Losonczy et al. (2003). Using this method, we obtained a mean inhibitory synaptic conductance (*g*_*i*_) of 2.33 ± 0.02 nS and excitatory conductance (*g*_*e*_) of 0.70 ± 0.01 nS. EPSCs and IPSCs were calculated by *I*_*i*_ = *g*_*i*_(*V* − *V*_*i*_) and *I*_*e*_ = *g*_*e*_(*V* − *V*_*e*_), respectively (*V*_*i*_ = −83 mV and *V*_*e*_ = 0 mV). Synaptic conductances were obtained by the differential equations: *dg*_*i*_/*dt* = −*g*_*i*_/τ_*i*_ and *dg*_*e*_/*dt* = −*g*_*e*_/τ_*e*_ with τ_*i*_ = 10 ms and τ_*e*_ = 5 ms.

The model was solved in real time using the 4th order Runge-Kutta method (with a *dt* = 0.05 ms) in a computer running Linux and the Real Time Application Interface (RTAI) from the Politecnico di Milano Institute-Dipartimento di Ingegneria Aerospaziale (Mantegazza, http://www.rtai.org/). Inputs to and outputs from the virtual neuron were acquired from the “10 Vm” outputs and the “command” inputs of the patch amplifiers as previously described (Leão et al., 2011). The order of simulations of different circuits was systematically varied to avoid statistical dependencies between the timing of recordings and the circuit investigated. Each PC pair responses to different circuit paradigms were recorded for 2 min with a 10 s delay between circuit protocols. Carbachol (10 μm, Sigma) was continuously added to the perfusate to increase the firing rate of PC to >2 Hz. In some experiments we used 10 μM ZD7288 (Tocris Cookson Inc., UK) to block the hyperpolarization-activated cyclic nucleotide-gated cation channel (HCN), 50 μM dAP5 (Sigma) to block NMDA receptors, 10 μM CNQX (Sigma) to block AMPA receptors and/or 10 μM picrotoxin (PTX, Sigma) to block GABA receptors.

### Data analysis

Matlab (version 2009a and 2011, Mathworks) was used for all data analysis. The first 30 s and the last 30 s of voltage traces were omitted for analysis to ensure circuit stability. Action potentials from PCs were detected based on threshold (> − 20 mV). Cross-correlation was computed by transforming spike trains in a series of 0s (no spike) and 1s (spike) with 0.1 ms-precision and computing the similarity between the binary sequences of the two PCs in each circuit as a function of time lag. Cross-correlograms (CCGs) were calculated using the “coeff” option of the cross-correlation command in Matlab (to scale the cross-correlation values from −1 to 1 and prevent dependency of the cross-correlation on the number of spikes) and then smoothed by a moving average filter with a span of 10 ms (Maex and De Schutter, 1998).

We examined cross-correlations over a lag range of ±1 s. Power spectral density analysis of binary spike series was made using Welch's method (“pwelch” command in Matlab). Synchrony index (SI) was defined as the maximum peak of the normalized CCG between −50 ms and 50 ms. This time window was chosen based on the analysis of the interspike intervals. The SI of the circuits was compared pair-wise using Student's *t*-test. Data is reported as mean ± Standard Error of the Mean (SEM).

As spike trains and synaptic events often show a high degree of nonlinearity (Leao et al., 2005), information theory algorithms were also applied to the analysis of spiking data. In that regard, cross-correlation describes the similarity of two signals but mutual information (Maex and De Schutter, 1998; Panzeri et al., 2007; Singh and Lesica, 2010) measures the statistical dependency between two discrete random variables (*X* and *Y*) without assuming linearity on this dependency (Maex and De Schutter, 1998; Panzeri et al., 2007; Singh and Lesica, 2010). The spike train sequences (binning = 20 ms) of the PCs (*pc*_left_ and *pc*_right_) can be described as features, whose actual values (0s, no spike and 1s, spike) are called feature values. The random variables (*PC*_left_ and *PC*_right_) formally describe the underlying probability structure (distribution) of a feature [*p*(*pc*_left_) and *p*(*pc*_right_)]. The overlap of the probability distributions of spike trains of each PC describes how the spike data from one of the cells can forecast the other. Hence, the individual entropies *H*(*PC*_left_) and *H*(*PC*_right_), calculated by:

$$H\left(PC_{\text{left}}\right)=-\sum_{pc_{\text{left}}}(p(pc_{\text{left}})*\log(p(pc_{\text{left}})))$$

describe the amount of information in each spike train sequence while joint entropy *H*(*PC*_left_, *PC*_right_):

$$H(PC_{\text{left}},PC_{\text{right}})=-\sum_{pc_{\text{left}}}\sum_{pc_{\text{right}}}(p(pc_{\text{left}},pc_{\text{right}})*\log(p(pc_{\text{left}},pc_{\text{right}})))$$

measures the amount of information in the combined trains of the two PCs *p*(*pc*_left_, *pc*_right_).

The mutual information *MI*(*PC*_left_, *PC*_right_) is then inferred as the sum of the two entropies *H*(*PC*_left_) and *H*(*PC*_right_) minus the joint entropy *H*(*PC*_left_, *PC*_right_), is calculated by:

$$\begin{array}{l} MI\left(PC_{\text{left}},PC_{\text{right}}\right)=MI\left(PC_{\text{right}},PC_{\text{left}}\right)\\ \;=H\left(PC_{\text{left}}\right)+H\left(PC_{\text{right}}\right)\\ \;-H(PC_{\text{left}},PC_{\text{right}}) \end{array}$$

and it quantifies the mutual dependence of the two PCs. In summary, the better the spiking activity of one PC can describe the spike activity of the other PC, the higher the mutual information between the two PCs.

In addition to MI, nonlinear relationships between PC spike data were also quantified by incremental mutual information (IMI) (Singh and Lesica, 2010). IMI is a method based on information theory and relies on decreasing the uncertainty (by of a time series *X* to its minimum before analysing the influence of another series *Y* in *X* (Singh and Lesica, 2010). For the computation of IMI, the entropies of conditioned (corrected for nontemporal dependencies) *X* and *Y* series verses delays of interest (in our case; spikes) are computed. Subsequently, the influence of *Y* over *X* at given delays is estimated while reducing the entropy due to *Y* conditioning (moving the time series forward or backward) around a given delay (Singh and Lesica, 2010). Singh and Leica have demonstrated that IMI is not only useful in showing if the association of two neurons arise from their connection or from a connection to a “third” pacing cell. Besides, IMI can also measure the strength and delays of connections (Singh and Lesica, 2010). Details on the computation of IMI can be found in the original publication and Matlab routines for its calculation were obtained from the authors' website (Singh and Lesica, 2010).

## Results

**Figure 1:**
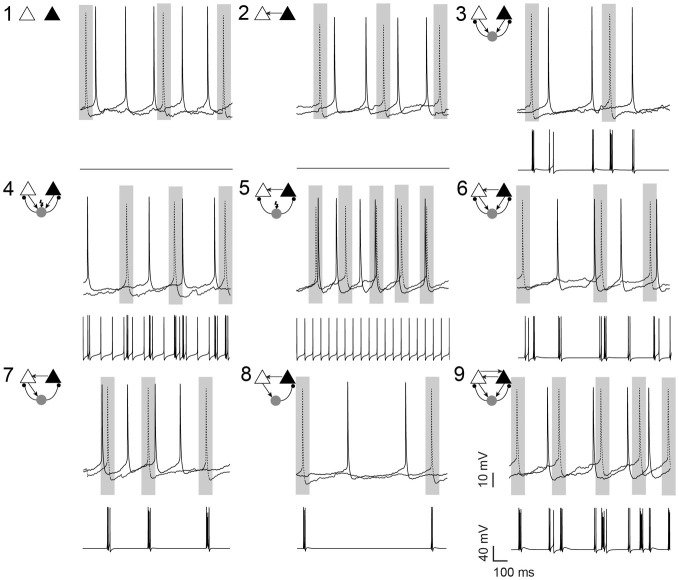
**Pyramidal cell firing in response to different neuronal circuit configurations.** Examples of current clamp recordings from a pyramidal cell pair (in the presence of 10 μM carbachol) and the changes in membrane potential of the simulated interneuron in different circuit configurations, as shown by the schematic insets. Shaded areas illustrate the 100 ms window where the synchrony index was calculated. In 7 circuits, a simulated fast-spiking interneuron (gray circle) formed hybrid microcircuits with or without excitatory (arrows) and inhibitory (small circles) connections. In circuits 4 and 5, current is applied to the simulated interneuron to generate spontaneous firing (flash symbol). Gray rectangles highlights 100 ms intervals centered at AP peaks of the left pyramidal cell (dotted line). Scale bars 10 mV, 100 ms.

**Figure 2:**
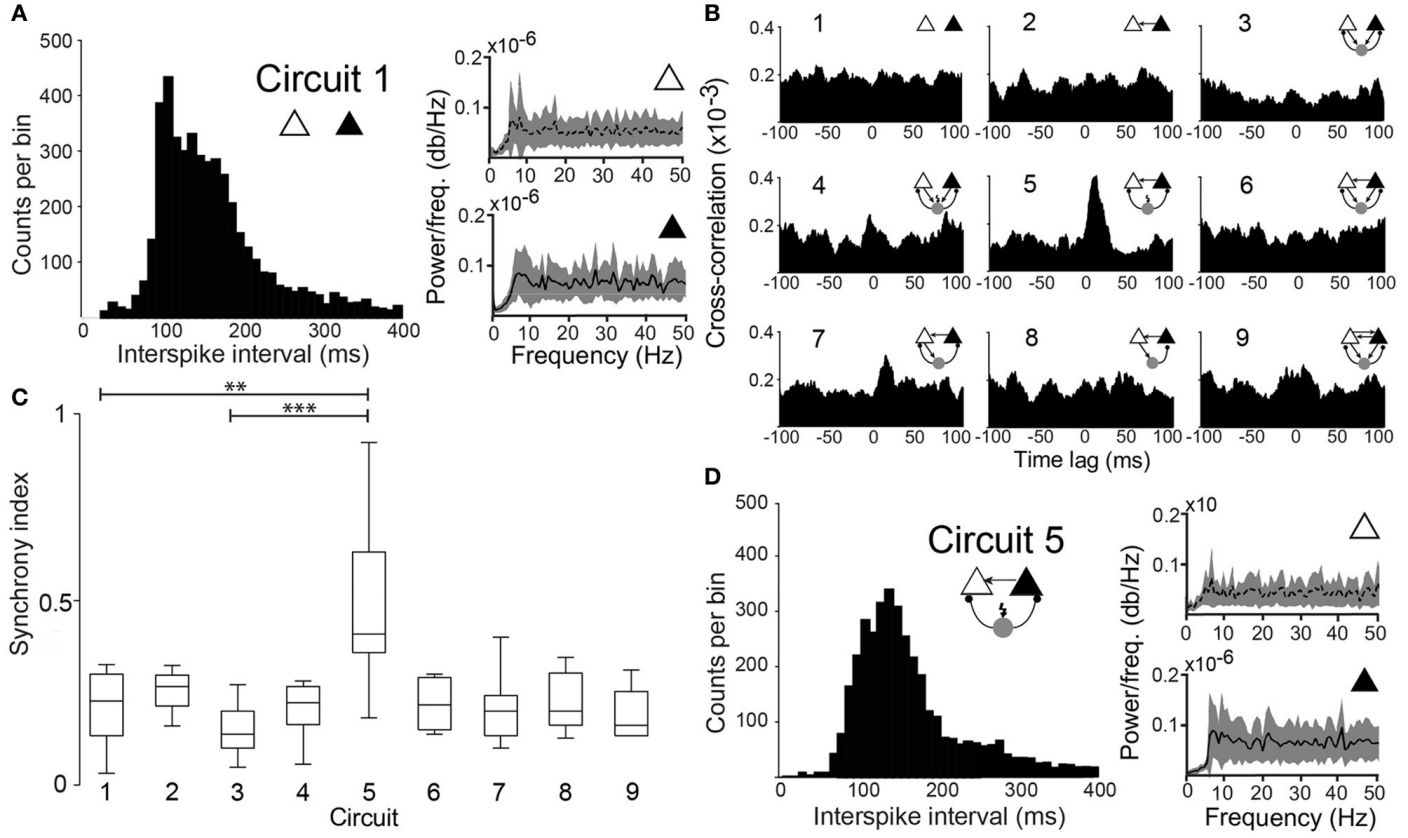
**Degree of synchronization depends on the circuit connectivity. (A)** Histogram of interspike intervals for circuit 1 (unconnected PCs). Power spectral density plots of the two PCs. **(B)** Mean cross-correlograms for the 9 different circuit schemes. **(C)** Box plots of the SI for the nine circuits tested shows statistical significant difference between circuit 1 and circuit 5 (*p* = 0.003, paired *t*-test, *n* = 12 pairs) and circuit 3 and circuit 5 (*p* = 0.0001, paired *t*-test, *n* = 12 pairs). (^**^p < 0.01, ^***^p < 0.001). **(D)** Same as in “A” for (Circuit 5).

We recorded from a total of 53 CA1 PC pairs connected or not connected to a virtual interneuron according to the connection diagrams shown in Figure 1. To assure that PC cells within the pair were drawn from a single population, we only used neurons with membrane properties (input resistance, cell capacitance, resting membrane potential and firing threshold) differing less than 10% from each other. Left and right PCs had a mean distance of 210 ± 52 μm and an average input resistance of 152.1 ± 1.9 and 151.0 ± 2.2 MΩ, respectively (*n* = 106, *p* = 0.72). Mean membrane capacitance was 48.8 ± 0.92 and 48.2 ± 1.2 pF (*n* = 106, *p* = 0.69), spike threshold (defined as the membrane voltage when the 1st derivative of the membrane potential reached values between 15 and 20 mV/ms) was −40.4 ± 0.9 and −39.9 ± 0.9 mV (*n* = 106, *p* = 0.66) and resting potential was −60.9 ± 1.1 and −58.7 ± 1.1 mV (*n* = 106, *p* = 0.16), respectively. There was no relationship between SI and inter PC distance. A spike of the virtual interneuron produced an IPSC at either or both PCs, depending on the connection scheme, with varied amplitudes (Maccaferri et al., 2000). Conversely, when connected to the virtual neuron or to each other, PC spikes elicited EPSCs with varied amplitudes in the postsynaptic cell (Maccaferri et al., 2000, see Methods). To assure that the two PCs were not natively interconnected, we injected current pulses to each PC separately and verified the lack of postsynaptic response on the other PC. Due to the low instantaneous firing rate (<1 Hz), we routinely added 10 μM carbachol to the aCSF to increase spiking activity. In the presence of carbachol, the average PC interspike interval was 278 ± 116 ms (*n* = 24, Figure 2A). In some circuits, the virtual interneuron fired APs at around 19 Hz due to the injection of external currents (Figure 1).

### Nonreciprocal synaptic connections favor synchrony

When unconnected, PCs produced nonregular and independent firing patterns evidenced by flat power spectral density plots and cross-correlograms (CCG) (Figures 2A,B, respectively). Coupling the two PCs nonreciprocally with an excitatory connection “Circuit 2” did not increase coordination (Figures 1, 2B,C) when compared to the unconnected pair “Circuit 1”. EPSCs alone, from the virtual synapses between PCs, were insufficient to produce APs in the postsynaptic cell. Connecting the two coupled PCs to a continuously firing virtual interneuron (~19 Hz, “Circuit 5”, Figure 1) caused a peak at (8.69 ± 0.80 ms) in the CCG, indicating PC synchronization (Figures 1, 2B,C). The amount of synchronization was estimated by formulating a synchronization index (SI) extracted from CCGs (see Methods). Mean SI of “Circuit 1” was equal to 0.27 ± 0.03 compared to a SI of 0.60 ± 0.07 in “Circuit 5” (*n* = 12, *p* = 0.003, paired *t*-test), suggesting that rhythmical inhibition associated to a unilateral connection between PCs favors synchronization. Interestingly, feedback connections from the PCs to the interneuron “Circuit 3” produced an unsynchronized network (SI = 0.19 ± 0.03, *n* = 12, not significantly different from “Circuit 1” and *p* = 0.0001 when compared to “Circuit 5”, paired *t*-test, Figures 2B,C). Of note, the baseline cross-correlation of two PCs in our experiments was 0.20 × 10^−3^ ± 1.16 × 10^−6^. That value could, therefore, be considered the chance level of the similarity of two randomly chosen, unconnected PCs. Despite the substantial increase in synchronization, mean interspike time in Circuit 5 of PCs (312 ± 140 ms, *n* = 24) remained unaltered when compared to “Circuit 1”, with PCs firing with no apparent rhythmicity (Figure 2D). We also found no significant difference between mean interspike times between the two PCs in “Circuit 5”. A similar behavior is found in CA3 PCs and basket cells during *in vitro* gamma oscillations: highly rhythmical basket cells with sparsely firing and PCs with no apparent rhythm) (Gulyás et al., 2010).

**Figure 3:**
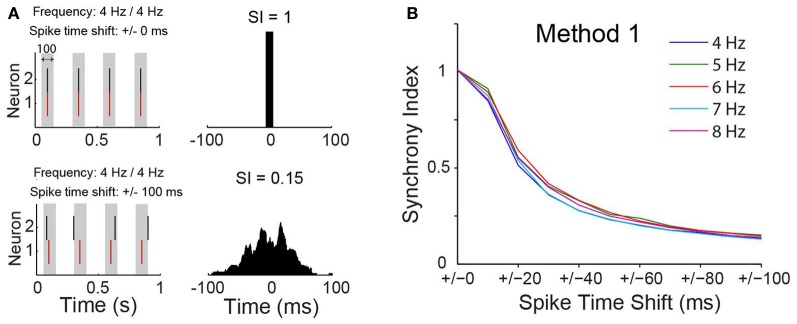
**Synchrony index has little dependence on spike frequency. (A)** Spike time of two simulated neurons without or with normally distributed pseudorandom shifts on spike times of one of the neurons. Gray box indicate a time window of 100 ms. Cross-correlograms for each case are shown on the right with value of the synchrony index (SI) indicated. **(B)** Relationship between SI and spike time shift when neurons are firing at different frequencies.

A concern using cross-correlation to measure synchrony across experiments is the spurious effect different firing frequency could have on estimates of synchrony. We calculated, therefore, the SI for synthesized spike data with varied firing frequencies following the addition of normally distributed pseudorandom time shifts to spike times (Figure 3A). We opted to use the maximum peak of the CCG normalized by the window size (see Materials and Methods). We have also considered a SI based on the area of the CCG or by normalizing the peak by spike counts. Nevertheless, the SI computation as described in the methods produced the least dependency on spike frequency (Figure 3). The SI decayed exponentially (Figure 3B) as a function of the amount of shifting, independent of spike frequency. This tendency was also observed when firing rates of the two model neurons were equally increased (Figure 3B). Hence, the SI described here is minimally affected by firing rate.

### Nonreciprocal connections increase pairwise mutual information of pyramidal cells

**Figure 4:**
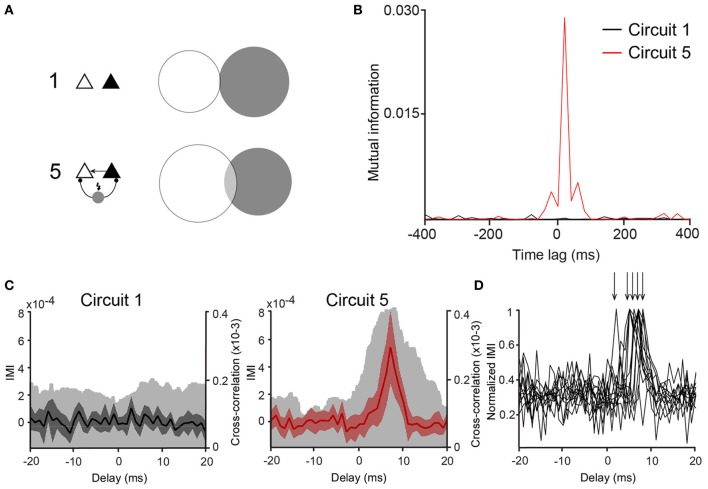
**Mutual information between PCs is larger in Circuit 5. (A)** An example of a Venn diagram showing mutual information (MI) as the degree of overlap of the two circles representing the entropy of two representative spike trains from two PCs from (Circuit 1) and (5). **(B)** Mutual information vs. time lag for the same two cases as in “**A**” shows high MI for circuit 5 (red trace) that is largest around lag 0. **(C)** Plots of Incremental Mutual Information (IMI) vs. delay of each spike compared to the next spike for (Circuit 1) and (5) (see Materials and Methods). (mean and 95% confidence interval, Cross-correlograms from Figure 2B are plotted for comparison—clear gray shades). **(D)** Normalized IMI vs. delay, arrows indicate peaks of individual cases.

Once the connection scheme generating the most synchrony among PCs was determined, concepts of information theory were applied to analyse whether the spiking activity of one of the PCs could predict the other. CCGs assume a linear dependency between the spiking of the two PCs, but overlook nonlinear relationships (Maex and De Schutter, 1998; Panzeri et al., 2007). Mutual information (MI) does not assume linearity in the relationship between two datasets and, therefore, it can complement CCGs when measuring spike synchrony. Hence, we have also computed if the connection scheme of “Circuit 5” affects mutual information (MI) between the spike data of the two PCs. We used binary spike data sampled at 20 ms bins as shorter periods would generate an amount of “0's” much larger than “1's”, decreasing drastically individual entropies while increasing the joint entropy in an unproportional fashion, producing spurious MI measurements (Butte and Kohane, 2000; Nirenberg and Latham, 2003). The pairwise MI of two unconnected PCs (*PC*_left_ and *PC*_right_) was MI = 0.0002 ± 0.0002 (n = 53) while in “Circuit 5” MI was equal to 0.0015 ± 0.0004 (n = 53, p = 0.002, paired *t-test*) (Figures 4A,B). Similarly to CCG data, other PC/virtual neuron circuits did not show a MI greater than “Circuit 1” (unconnected PCs).

Whilst there were no explicit delays added to virtual synapses, delays could arise implicitly due to changes in membrane time constant, availability of Na^+^ channels or heterogeneity of ion currents or the balance between excitation and inhibition (Leao et al., 2006b; Leão et al., 2011). Mutual information between two spike train sequences *PC*_left_ and *PC*_right_ was, therefore, calculated as a function of the time lag *MI*[*PC*_left_ (*t*), *PC*_right_(*t* − *dt*)]. MI peaks were, in some cases, found when *dt* = 20 ms (14 out of 53 pairs). We further investigated the appearance of functional delays by computing the IMI of the spike data as described in Singh and Lesica (2010) using binary spike data 2 ms bins. Out of the 53 pairs, 12 pairs showed statistically valid IMI measurements (see Singh and Lesica, 2010). No evident peaks in “Circuit 1” but peaks at various delays (averaging 6 ± 0.5 ms, *n* = 12, Figures 4C,D) were detected. These results suggest that the influence of the cells to each other in “Circuit 5” is noninstantaneous and a rather functional delay in the connections is being generated by either membrane currents (Leao et al., 2006b) or the balance of excitation/inhibition (Leão et al., 2011).

### *I*_*h*_ contributes to pyramidal cell spiking synchrony in “Circuit 5”

**Figure 5:**
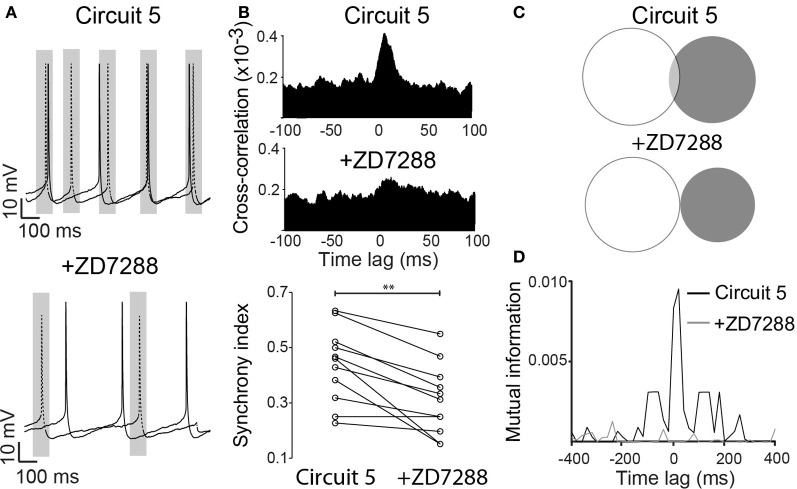
**ZD7288 disrupts synchronization in a CA1 artificial neuronal circuit. (A)** Representative traces of action potentials from the two PCs connected in (Circuit 5) before and after bath application of ZD7288 (10 μM). Gray box indicate a 100 ms interval around on of the PC spikes as in Figure 1. Scale bars 10 mV, 100 ms. **(B)** Mean cross-correlograms for circuit 5 before and after ZD7288. Summary of the synchrony index for Circuit 5 before and after ZD7288 (^**^p = 0.002, *n* = 11 pairs). **(C)** Venn diagrams of mutual information (MI) from traces in “**A**” before and after ZD7288. **(D)** Plot of MI vs. Time lag for (Circuit 5) before and after ZD7288.

*I*_*h*_ exert a strong influence on the response of the PCs to synaptic inputs (Magee, 1999), and it could consequently affect the synchronization of spike activity. To test this hypothesis, we blocked *I*_*h*_ by bath application of ZD7288 (10 μM) with the “Circuit 5” connection scheme. In the presence of ZD7288, the SI of “Circuit 5” decreased to 0.31 ± 0.04 compared to 0.44 ± 0.04 prior to the application of the drug (*n* = 11, *p* = 0.002, paired *t*-test, Figures 5A,B) and blocking *I*_*h*_ almost flattened the CCG (Figure 5B). Mutual information showed a more dramatic decrease after the application of ZD7288. An initial MI = 0.0028 ± 0.0009 of “Circuit 5” at 0-lag dropped almost 10-fold to 0.0003 ± 0.0001 (*n* = 11, p = 0.011, Figures 5C,D). The addition of an artificial *I*_*h*_ (dynamic clamp, using the fast *I*_*h*_ model from Leao et al. (2006a,b) was not sufficient to reset neither the SI or the MI values observed previously to the application of ZD7288 (data not shown). Taken together, this data suggests that *I*_*h*_ contributes to the synchronization observed in “Circuit 5”. This effect, however, is not generated by HCN present in perisomatic compartments (where the virtual connections are implemented through a point process) suggesting that dendritic *I*_*h*_ is also important for the appearance of PC coordination in “Circuit 5”.

### NMDA receptors influence pyramidal cell synchronization

**Figure 6:**
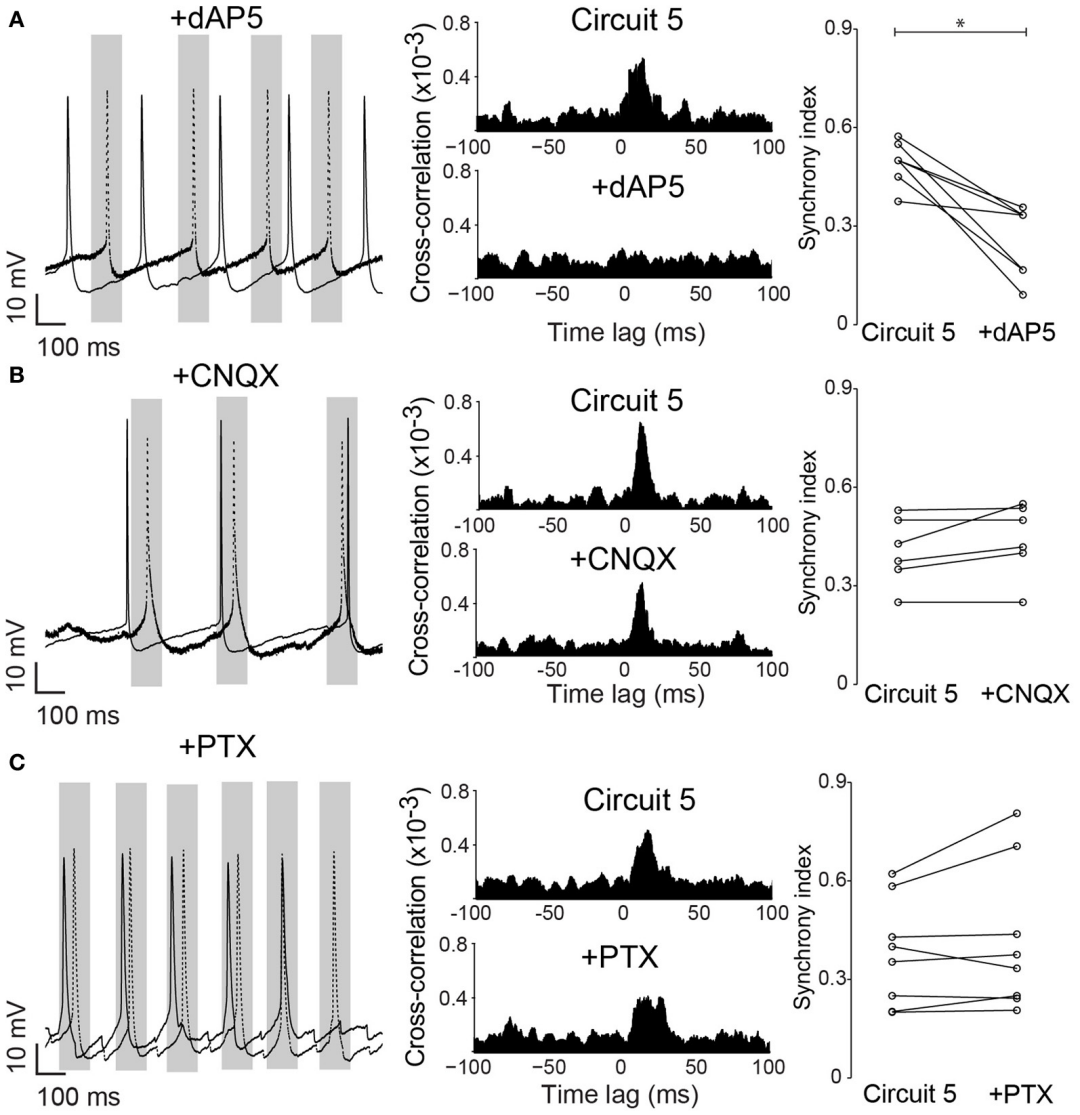
**Native NMDA receptors are important for pyramidal cell synchronization in a hybrid CA1 neuronal circuit. (A)** *left*, Example traces from (Circuit 5) after bath application of the NMDA receptor blocker dAP5 (50 μM); *middle*, Mean cross-correlograms of (Circuit 5) before and after dAP5; *right*, Summary of SI for each pair of PCs before and after dAP5 (^*^p = 0.03, *n* = 9). **(B)** *left*, Example traces from circuit 5 before and after application of the AMPA receptor blocker CNQX (10 μM) application; *middle*, Mean cross-correlation plots of Circuit 5 before and after CNQX; *right*, Summary of SI for each pair of PCs before and after CNQX (*p* = 0.66, *n* = 6). **(C)** *left*, Example traces from Circuit 5 before and after application of the GABA receptor blocker PTX (10 μM); *middle*, Mean cross-correlation plots of circuit 5 before and after PTX; *right*, Summary of SI for each pair of PCs before and after PTX (10 μM) (*p* = 0.43, *n* = 8). Gray box shows a time interval of 100 ms around one of the PCs firing (dotted line). Scale bars 10 mV, 100 ms.

**Figure 7:**
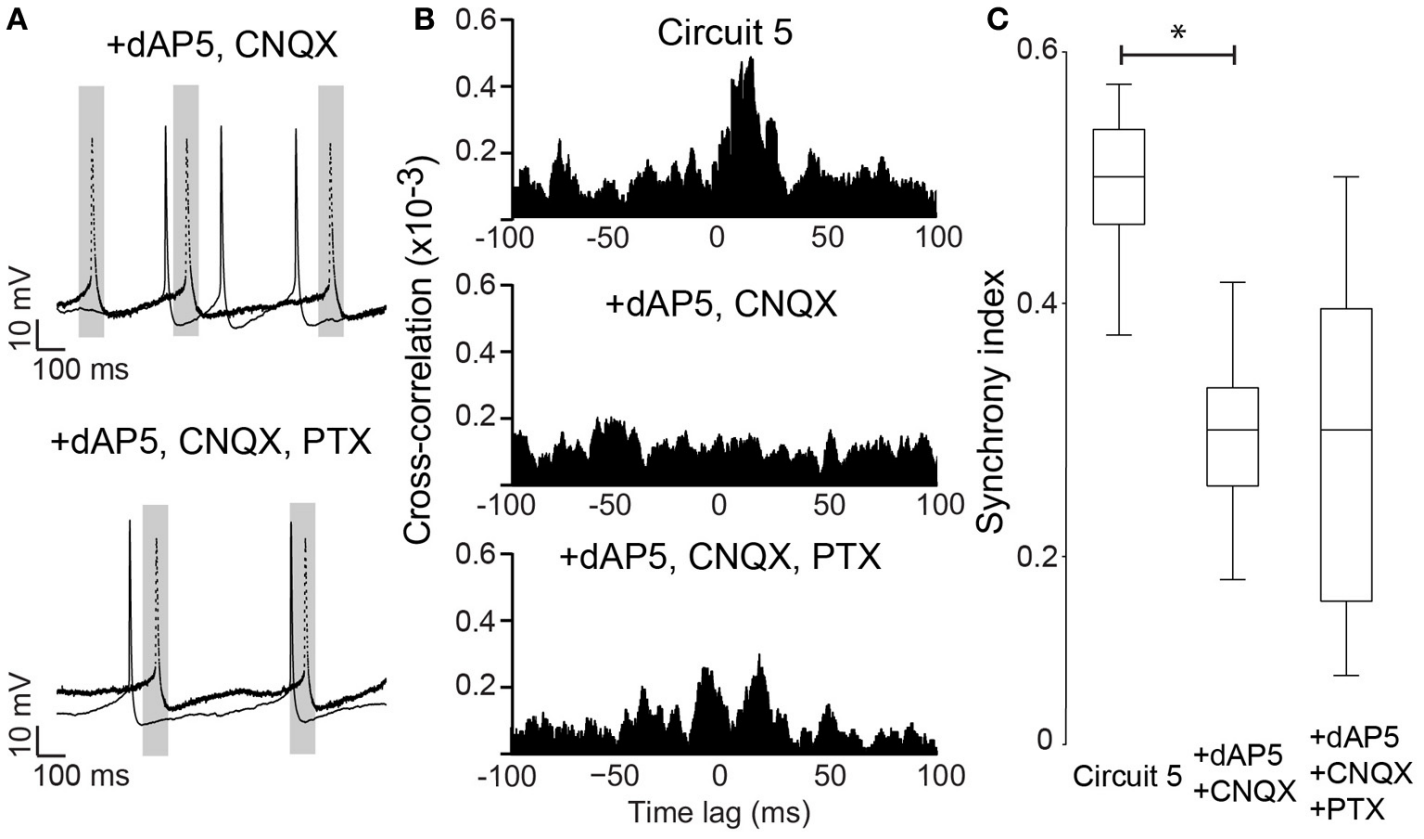
**NMDA block alone is sufficient to disrupt synchrony in Circuit 5. (A)** *Top*, Example traces from the two PCs in (Circuit 5) in the presence of both dAP5 (50 μM) and CNQX (10 μM). *Bottom*, The same cell recordings as in “**A**” with the further application of PTX (10 μM). Gray box shows a 100 ms interval around spikes form one of the PCs (dotted line). Scale bars: 10 mV, 100 ms. **(B)** *Top*,**** Mean Cross-correlograms of spike trains from circuit 5 without synaptic blockers; *middle*, in the presence of dAP5 and CNQX; *bottom*, in the presence of dAP5, CNQX and PTX. **(C)** Box plots of mean SI (^*^p = 0.04).

Neuronal excitability of a neuron is not only influenced by voltage-gated or leakage channels. Sustained synaptic currents have additionally a strong influence on the neuron's transfer function (Wolfart et al., 2005). Therefore, we assessed the effect of background synaptic activity on PCs by applying AMPA, NMDA and GABA receptor blockers (CNQX, dAP5 and PTX, respectively) to the perfusate while maintaining the connection scheme of “Circuit 5”. When synaptic blockers were applied individually, CNQX and PTX showed no effect on SI (*n* = 6 and 8, respectively, Figure 6). Application of PTX has, however, decreased the interspike interval in the absence of a holding current (from 364 ± 116 ms to 206 ± 93 ms, *n* = 8, *p* = 0.006, paired *t*-test, two-tailed). When dAP5 was present, the SI of “Circuit 5” decreased from 0.50 ± 0.02 to 0.26 ± 0.04 (*n* = 9, *p* = 0.03, paired *t*-test, Figure 6A). Also simultaneous application of dAP5+CNQX decreased the SI of “Circuit 5” (from 0.50 ± 0.02 to 0.30 ± 0.03, *n* = 9, *p* = 0.04, paired *t*-test) (Figure 7) but not differently from individual application of dAP5. There was no significant difference of SI (compared to the control condition) found if PTX was further added to the perfusate (*n* = 7, Figure 7). Interestingly, the interspike interval increased compared to control conditions (from 364 ± 116 ms, *n* = 8 to 492 ± 182 ms, *n* = 7, *p* = 0.01, paired *t*-test, two-tailed) or PTX alone (*p* = 0.004, paired *t*-test, two-tailed). These results indicate that, background synaptic activity indeed contributes to setting the neuronal transfer function and, therefore, the synchronization tendency of a microcircuit.

## Discussion

Here, we have combined paired patch clamp recordings with realtime simulation of an interneuron and synaptic connections to build hybrid hippocampal microcircuits. The purpose of this work was to assess how different connection parameters contribute to spike synchronization of pyramidal cells (PC). We found that the circuit in which PCs were unidirectionally connected and the virtual interneuron supply inputs to both PCs without receiving feedback (Circuit 5 in Figure 1) was the most synchronous. Pharmacological block of HCN channels dramatically affected the synchronicity of this network. In addition, external synaptic activity had a substantial contribution to PC synchronization. When NMDA receptors were blocked we observed a drastic decrease of the synchronized PC activity, however, no effect on PC synchronization was observed when AMPA receptors or GABA receptors were blocked. Synchronization of firing is encountered during several cognitive processes and defines Hebb's assemblies (Hebb, 1949; Lopes-dos-Santos et al., 2011; Zullo et al., 2012). Hebb's postulated that when a group of neurons fire together their united activity facilitates the action of each other (Hebb, 1949). Coordinated activity increases the representation and the efficiency of a stimulus in the postsynaptic target (Mandairon et al., 2006). It was previously thought that synchronization arises from interacting excitatory cells. However, in model studies synchronization through excitation is mostly featured in systems with no synaptic delays and, when delays are present, inhibitory synapses are more suitable to generate synchrony (Van Vreeswijk et al., 1994). We showed that the microcircuit configuration generating the largest amount of synchrony between the PC pair consisted of an interneuron firing independently from the PCs and a unidirectional connection between the PCs (Circuit 5). As the simulated interneuron fires independently from the PC in Circuit 5, changes in IPSC delays (in relation to the interneuron spike) would not affect PC synchronization. We did not investigate the importance of conduction delays in circuits where feedback from PCs was present. Nevertheless, our connection schemes have generated functional delays that were probably an effect from the state of ionic currents at the membrane or the balance between excitation and inhibition. A similar phenomenon was observed in the calyx of Held synapse, where a combination of different densities of K^+^ and HCN channels created functional synaptic delays independent of axial resistance and conduction velocity (Leao et al., 2006b).

Synchronization through feedback inhibition has been shown in several works and it seems to be responsible to the generation of rhythms like gamma oscillations. However, in our simplified circuit of feedback inhibition (Circuit 3) failed to produce synchronization. A reason for that could be due to the reduced amount of excitatory afference to the interneuron (only 2 PCs). In real biological circuits, basket cells can receive excitation of thousands of PCs and drive the rhythm of a given PC with little dependence from that PC firing. It will be interesting to investigate in the future a circuit similar to Circuit 3 in which the interneuron receives either a constant current to drive rhythmical firing or background synaptic activity, causing the generation of spikes in the interneurons uncorrelated to PC spikes. Other circuits like reciprocally connected PCs without interneuron connections could have been also explored. However, there is no description of such configuration in hippocampal or neocortical circuits and to maintain the quality of our experimental preparation we had to limit the recording times and, therefore, explored only a limited number of circuit configurations (Figure 1). Basket cells exhibit divergent connections to PCs, which in turn share a common individual interneuron (Sik et al., 1995). Hence, excitation provided by PCs to basket cells would appear to be tonic (Sik et al., 1995; Williams and Kauer, 1997; Tort et al., 2007; Gulyás et al., 2010) while basket cells supply PCs with phasic inhibition (Cobb et al., 1995; Gulyás et al., 2010). Thus forming a mesoscopic circuit analogous to the “Circuit 5” case. Interestingly, in “Circuit 5”, PC spikes were not rhythmical despite being synchronized with longer interspike intervals than the simulated interneuron. This firing behavior is often observed during gamma oscillations but poorly reproduced in purely mathematical models (Williams and Kauer, 1997; Mann et al., 2005; Leão et al., 2009). As a large number of PCs converge their outputs to basket cells, there is little spike locking between individual PCs and a basket cell. Individual basket cells can through divergent connections, however, drive the spike time of a group of PCs (Cobb et al., 1995).

The recurrent excitation implemented in “Circuit 5” is common in neocortical microcircuits (Silberberg and Markram, 2007) and, while not as common in CA1, there are a number of studies demonstrating their existence (Crépel et al., 1997; Aniksztejn et al., 2001; Fink et al., 2007). Feedback connections among CA1 PCs are prevalent in the developing brain but are rarely found in the adult hippocampus (Crépel et al., 1997). Deuchars and Thomson (1996) reported connections between CA1 PC in about 1% of their recorded pairs. Blocking inhibition, however, increased the likelihood of finding PC to PC connections in CA1 (Crépel et al., 1997; Fink et al., 2007). Lesions using kainate in rats also promote CA1 the appearance of PC connection recurrence (Perez et al., 1996). Feedback CA1 PC synapses are also special in terms of synaptic plasticity and activity history can convert CA1 from a parallel circuit to a network with sparse recurrence (Fink et al., 2007). Hence, the connection scenario of “Circuit 5” is plausible in both neocortex and hippocampus (Cobb et al., 1995; Silberberg and Markram, 2007). Important to note in hippocampal networks, long-lasting synchrony cannot be produced when the representation of PC inputs onto basket cells is increased (by coordinated firing of a large number of PCs) (Whittington et al., 1997; Traub et al., 1999; Leão et al., 2009).

Coordination of PC activity by basket cells is also believed to be essential for the generation of gamma oscillations (Mann et al., 2005). Albeit not necessarily being the cause of local field potential (LFP) generation, the emergence of LFP is often followed by increased firing synchrony in the vicinity of the recording site (Nauhaus et al., 2009; Denker et al., 2011). Similarly to our results, increased synchrony of PC spiking during rhythmical activity such as gamma oscillations is not necessarily translated to rhythmical firing of PCs (Williams and Kauer, 1997; Tort et al., 2007; Leão et al., 2009). In fact, during gamma oscillations, PC APs are weakly locked to the oscillatory process, despite the high coherence of subthreshold changes in PC membrane potential and the LFP (Williams and Kauer, 1997; Tort et al., 2007; Leão et al., 2009). On the other hand, basket cells fire APs at every cycle of a gamma oscillation in a phase-locked manner (Mann et al., 2005; Gulyás et al., 2010). Nevertheless, the induction of gamma oscillations requires an increase in PC synchronization (Tort et al., 2007) and increasing AP frequency (decreasing the coherence between PC firing and the oscillatory LFP) which dramatically reduces LFP amplitude. Despite the increased drive from PC to interneurons when PCs are overexcited (for example, when resting K^+^ currents are blocked, Leão et al., 2009), gamma oscillations still decrease, suggesting that a phasic feedback to basket cells may indeed be deleterious to the generation of gamma oscillations.

In addition to connectivity patterns, we investigated the influence of the intrinsic current *I*_*h*_. *I*_*h*_ is a major determinant of resonance frequency and input resistance of CA1 PCs (Hu et al., 2002). Spike time in response to incoming excitation or inhibition is largely affected by *I*_*h*_ as this current shortens EPSPs and shapes rebound spiking (Leao et al., 2006a; Gastrein et al., 2011). It is, therefore, expected that this current has a major effect on the PC responses to synaptic inputs and, consequently, to PC synchronization. In accordance, blocking *I*_*h*_ led to reduced PC synchronization. Interestingly, artificially injecting *I*_*h*_ currents in the PC soma after ZD7288 did not recover synchronization. This result may be explained by the fact that the net effect of *I*_*h*_ on cell excitability and spike timing is closely related to its compartmentalization. Namely, dendritic *I*_*h*_ favors coincidence detection and low excitability while somatic *I*_*h*_ favors increased excitability and temporal summation (Santoro and Baram, 2003; Leão et al., 2011). In auditory neurons, for example, somatic *I*_*h*_ has the largest influence on rebound spiking when compared to dendritic *I*_*h*_ (Leão et al., 2011). *I*_*h*_ is inhomogeneously distributed along PC dendrites (Magee, 1999). In our experiments, simulated *I*_*h*_ and synaptic currents were applied directly in the PC soma and, therefore, we cannot infer about the influence of distal synapses and dendritic *I*_*h*_ on PC synchronization. Dendritic *I*_*h*_ is likely to affect background synaptic currents arising on the dendrite. For example, blocking *I*_*h*_ in a subcellular compartment can dramatically increase the input resistance of that compartment, increasing the change in potential generated by an EPSC or IPSC (Leão et al., 2011). In other words, as the membrane time constant becomes larger after *I*_*h*_ blockage, PCs will fire more indiscriminately to synaptic inputs (Leão et al., 2011), decreasing the relevance of synchronous synaptic currents.

We have used carbachol to increase PC firing and, consequently, the excitability of all networks in the slice. Hence, PCs received a large number of randomly occurring synaptic inputs, both excitatory and inhibitory. In a model of spinal cord circuits, the addition of background synaptic activity decreased spike synchronization and firing frequency, independently of the incoherent synapse nature (AMPA or NMDA) (Kohn, 1998). There are also computational works demonstrating that “optimal” synaptic background levels improve synchronization (Balenzuela and García-Ojalvo, 2005; Perc, 2009). This effect, however, was not shown in biological systems. We found that the blocking of AMPA or GABA synapses did not affect synchronization but NMDA blockage strongly decreased it. We hypothesize that one of the effects of NMDA synapses on synchronization could arise from the fact that somatic depolarization invading the dendrite could coincide with tonic postsynaptic activation of dendritic NMDA receptors. Activation of these synapses would fail to produce postsynaptic changes in potential except in the presence of postsynaptic depolarization. Thus, tonic NMDA activation would promote the amplification of incoming excitation, facilitating the generation of spikes, and, consequently, synchronization. In addition, NMDA receptors, like *I*_*h*_, are unevenly distributed in the somatodendritic axis and it can also alter the input resistance of dendritic compartments (Kim et al., 2012). Hence, the blockade of NMDA receptors could also affect synchrony by affecting the membrane time constant of the dendrites.

As it was not in the scope of the present work to study the role of interneuron properties on PC synchronization, we opted to use a simplified interneuron model (Wang and Buzsáki, 1996). The simplicity of the model allowed us to perform fast calculations (20 kHz) required for real time interactions. The modeled interneuron is able to reproduce some features of basket cells, such as tonic high frequency firing and low firing adaptation (Aponte et al., 2006), and has been used to show that gamma oscillations can arise from purely inhibitory networks (Wang and Buzsáki, 1996). However, the lack of *I*_*h*_ or Ca^2+^ currents in the model could lead to different integration of synaptic inputs and spiking regularity than in actual basket cells (Aponte et al., 2006). Besides that, the high input resistance [132.50 MΩ compared to approximately 80–100 MΩ in real basket cells (Aponte et al., 2006)] of the modeled cell leads to the generation of APs for most presynaptic APs. Nevertheless, interneuron models with more realistic input resistance (Aponte et al., 2006) would not spike in our hybrid network since the model interneuron only received inputs from 2 PCs.

In summary, we have shown that nonreciprocal excitation promotes synchronization only when associated with tonic inhibition in CA1 microcircuits. It is important to point out that IPSCs originating from basket cells in real networks target perisomatic compartments of PCs (Sik et al., 1995), making our approach (injected IPSCs on PC somas through the patch pipette) realistic. We have proposed several connection diagrams for a minimal hippocampal microcircuit aiming to find the configuration most prone to produce synchrony. Our results demonstrated that in a triple cell circuit (2 PCs and 1 basket cell), not inhibition or excitation alone but a combination of the two promotes synchrony. The largest synchronization observed in “Circuit 5” compared to other architectures could be due to the nonstationary firing rate of PCs after carbachol application and the nondeterministic nature of AP generation after a pre-synaptic spike. Simulated EPSCs could not produce postsynaptic APs *per se*, explaining the low synchronization in circuits comprised solely by inter PC connections. However, IPSCs arising from interneurons could organize the excitatory drive from other sources, generating “windows of opportunities” for the simulated EPSP to increase the probability of a postsynaptic AP (Engel et al., 2001). Besides, as the membrane potential is largely depolarized by the presence of carbachol, IPSPs could also help to lift Na+ channels from their inactive state, facilitating AP generation after the inhibition decays. Nevertheless, in real hippocampal circuits, there is a blend of connection patterns between PCs and different classes of interneurons, which can also interconnect (Leão et al., 2012). Hence, while the connection pattern of “Circuit 5” may produce the largest synchronicity in our hybrid circuit, the interaction of all the elements in hippocampal networks may require other network architectures to produce synchrony. It will be interesting in the future to add more and different types of interneurons to the network in order to understand how these elements interact with real PCs.

### Conflict of interest statement

The authors declare that the research was conducted in the absence of any commercial or financial relationships that could be construed as a potential conflict of interest.

## References

[B1a] Abeles M. Prut Y. Bergman H. Vaadia E. (1994). Synchronization in neuronal transmission and its importance for information processing. Prog. Brain Res. 102, 395–404 10.1016/S0079-6123(08)60555-5 7800829

[B1] Aniksztejn L. Demarque M. Morozov Y. Ben-Ari Y. Represa A. (2001). Recurrent CA1 collateral axons in developing rat hippocampus. Brain Res. 913, 195–200 10.1016/S0006-8993(01)02817-7 11549387

[B2] Aponte Y. Lien C.-C. Reisinger E. Jonas P. (2006). Hyperpolarization-activated cation channels in fast-spiking interneurons of rat hippocampus. J. Physiol. (Lond.) 574, 229–243 10.1113/jphysiol.2005.104042 16690716 PMC1817792

[B3] Bal T. McCormick D. A. (1997). Synchronized oscillations in the inferior olive are controlled by the hyperpolarization-activated cation current I(h). J. Neurophysiol. 77, 3145–3156 9212264 10.1152/jn.1997.77.6.3145

[B4] Balenzuela P. García-Ojalvo J. (2005). Role of chemical synapses in coupled neurons with noise. Phys. Rev. E Stat. Nonlin. Soft. Matter. Phys. 72:021901 10.1103/PhysRevE.72.021901 16196598

[B5] Butte A. J. Kohane I. S. (2000). Mutual information relevance networks: functional genomic clustering using pairwise entropy measurements. Pac. Symp. Biocomput. 418–429. 10902190 10.1142/9789814447331_0040

[B6] Cobb S. R. Buhl E. H. Halasy K. Paulsen O. Somogyi P. (1995). Synchronization of neuronal activity in hippocampus by individual GABAergic interneurons. Nature 378, 75–78 10.1038/378075a0 7477292

[B7] Colom L. V. (2006). Septal networks: relevance to theta rhythm, epilepsy and Alzheimer's disease. J. Neurochem. 96, 609–623 10.1111/j.1471-4159.2005.03630.x 16405497

[B8] Crépel V. Khazipov R. Ben-Ari Y. (1997). Blocking GABA(A) inhibition reveals AMPA- and NMDA-receptor-mediated polysynaptic responses in the CA1 region of the rat hippocampus. J. Neurophysiol. 77, 2071–2082. 9114256 10.1152/jn.1997.77.4.2071

[B9] Denker M. Roux S. Lindén H. Diesmann M. Riehle A. Grün S. (2011). The local field potential reflects surplus spike synchrony. Cereb. Cortex 21, 2681–2695 10.1093/cercor/bhr040 21508303 PMC3209854

[B10] Deuchars J. Thomson A. M. (1996). CA1 pyramid-pyramid connections in rat hippocampus *in vitro*: dual intracellular recordings with biocytin filling. Neuroscience 74, 1009–1018 8895869 10.1016/0306-4522(96)00251-5

[B11] Engel A. K. Fries P. Singer W. (2001). Dynamic predictions: oscillations and synchrony in top-down processing. Nat. Rev. Neurosci. 2, 704–716 10.1038/35094565 11584308

[B12] Fink A. E. Sariñana J. Gray E. E. O'Dell T. J. (2007). Activity-dependent depression of local excitatory connections in the CA1 region of mouse hippocampus. J. Neurophysiol. 97, 3926–3936 17409173 10.1152/jn.00213.2007

[B13] Gastrein P. Campanac E. Gasselin C. Cudmore R. H. Bialowas A. Carlier E. (2011). The role of hyperpolarization-activated cationic current in spike-time precision and intrinsic resonance in cortical neurons *in vitro*. J. Physiol. (Lond.) 589, 3753–3773 10.1113/jphysiol.2011.209148 21624967 PMC3171884

[B14] Gulyás A. I. Szabó G. G. Ulbert I. Holderith N. Monyer H. Erdélyi F. (2010). Parvalbumin-containing fast-spiking basket cells generate the field potential oscillations induced by cholinergic receptor activation in the hippocampus. J. Neurosci. 30, 15134–15145 10.1523/JNEUROSCI.4104-10.2010 21068319 PMC3044880

[B15] Hansel D. Mato G. Meunier C. (1995). Synchrony in excitatory neural networks. Neural Comput. 7, 307–337 10.1162/neco.1995.7.2.307 8974733

[B16] Hebb D. (1949). The Organization of Behavior: A Neuropsychological Theory. New York, NY: John Wiley and Sons, Inc

[B17] Hu H. Vervaeke K. Storm J. F. (2002). Two forms of electrical resonance at theta frequencies, generated by M-current, h-current and persistent Na+ current in rat hippocampal pyramidal cells. J. Physiol. (Lond.) 545, 783–805 10.1113/jphysiol.2002.029249 12482886 PMC2290731

[B18] Kim S. Guzman S. J. Hu H. Jonas P. (2012). Active dendrites support efficient initiation of dendritic spikes in hippocampal CA3 pyramidal neurons. Nat. Neurosci. 15, 600–606 10.1038/nn.3060 22388958 PMC3617474

[B19] Kohn A. F. (1998). Effects of synaptic noise on a neuronal pool model with strong excitatory drive and recurrent inhibition. Biosystems 48, 113–121 10.1016/S0303-2647(98)00056-2 9886638

[B20] Leao K. E. Leao R. N. Sun H. Fyffe R. EW. Walmsley B. (2006a) Hyperpolarization-activated currents are differentially expressed in mice brainstem auditory nuclei. J. Physiol. (Lond.) 576, 849–864 10.1113/jphysiol.2006.114702 16916913 PMC1890420

[B21] Leao R. N. Sun H. Svahn K. Berntson A. Youssoufian M. Paolini A. G. (2006b) Topographic organization in the auditory brainstem of juvenile mice is disrupted in congenital deafness. J. Physiol. (Lond.) 571, 563–578 10.1113/jphysiol.2005.098780 16373385 PMC1805808

[B22] Leão K. E. Leão R. N. Walmsley B. (2011). Modulation of dendritic synaptic processing in the lateral superior olive by hyperpolarization-activated currents. Eur. J. Neurosci. 33, 1462–1470 10.1111/j.1460-9568.2011.07627.x 21366727

[B23] Leao R. N. Leao F. N. Walmsley B. (2005). Non-random nature of spontaneous mIPSCs in mouse auditory brainstem neurons revealed by recurrence quantification analysis. Proc. Biol. Sci. 272, 2551–2559 10.1098/rspb.2005.3258 16271982 PMC1599776

[B24] Leão R. N. Mikulovic S. Leão K. E. Munguba H. Gezelius H. Enjin A. (2012). OLM interneurons differentially modulate CA3 and entorhinal inputs to hippocampal CA1 neurons. Nat. Neurosci. 15, 1524–1530 10.1038/nn.3235 23042082 PMC3483451

[B25] Leão R. N. Tan H. M. Fisahn A. (2009). Kv7/KCNQ channels control action potential phasing of pyramidal neurons during hippocampal gamma oscillations *in vitro*. J. Neurosci. 29, 13353–13364 10.1523/JNEUROSCI.1463-09.2009 19846723 PMC6665214

[B26] Lopes-dos-Santos V. Conde-Ocazionez S. Nicolelis M. A. L. Ribeiro S. T. Tort A. B. L. (2011). Neuronal assembly detection and cell membership specification by principal component analysis. PLoS ONE 6:e20996 10.1371/journal.pone.0020996 21698248 PMC3115970

[B26a] Losonczy A. Somogyi P. Nusser Z. (2003). Reduction of excitatory postsynaptic responses by persistently active metabotropic glutamate receptors in the hippocampus. J. Neurophysiol. 89, 1910–1919 10.1152/jn.00842.2002 12686572

[B27] Maccaferri G. Roberts J. D. Szucs P. Cottingham C. A. Somogyi P. (2000). Cell surface domain specific postsynaptic currents evoked by identified GABAergic neurones in rat hippocampus *in vitro*. J. Physiol. (Lond.) 524 Pt 1, 91–116 10.1111/j.1469-7793.2000.t01-3-00091.x 10747186 PMC2269850

[B28] Maex R. De Schutter E. (1998). Synchronization of golgi and granule cell firing in a detailed network model of the cerebellar granule cell layer. J. Neurophysiol. 80, 2521–2537 9819260 10.1152/jn.1998.80.5.2521

[B29] Magee J. C. (1999). Dendritic Ih normalizes temporal summation in hippocampal CA1 neurons. Nat. Neurosci. 2, 848 10461231 10.1038/12229

[B30] Mandairon N. Stack C. Kiselycznyk C. Linster C. (2006). Broad activation of the olfactory bulb produces long-lasting changes in odor perception. Proc. Natl. Acad. Sci. U.S.A. 103, 13543–13548 10.1073/pnas.0602750103 16938883 PMC1569199

[B31] Mann E. O. Suckling J. M. Hajos N. Greenfield S. A. Paulsen O. (2005). Perisomatic feedback inhibition underlies cholinergically induced fast network oscillations in the rat hippocampus *in vitro*. Neuron 45, 105–117 10.1016/j.neuron.2004.12.016 15629706

[B32] Nauhaus I. Busse L. Carandini M. Ringach D. L. (2009). Stimulus contrast modulates functional connectivity in visual cortex. Nat. Neurosci. 12, 70–76 10.1038/nn.2232 19029885 PMC2610236

[B33] Nirenberg S. Latham P. E. (2003). Decoding neuronal spike trains: how important are correlations? Proc. Natl. Acad. Sci. U.S.A. 100, 7348–7353 10.1073/pnas.1131895100 12775756 PMC165878

[B34] Paluszkiewicz S. M. Olmos-Serrano J. L. Corbin J. G. Huntsman M. M. (2011). Impaired inhibitory control of cortical synchronization in fragile X syndrome. J. Neurophysiol. 106, 2264–2272 10.1152/jn.00421.2011 21795626 PMC3214096

[B35] Panzeri S. Senatore R. Montemurro M. A. Petersen R. S. (2007). Correcting for the sampling bias problem in spike train information measures. J. Neurophysiol. 98, 1064–1072 10.1152/jn.00559.2007 17615128

[B36] Perc M. (2009). Optimal spatial synchronization on scale-free networks via noisy chemical synapses. Biophys. Chem. 141, 175–179 10.1016/j.bpc.2009.01.012 19232814

[B37] Perez Y. Morin F. Beaulieu C. Lacaille J. C. (1996). Axonal sprouting of CA1 pyramidal cells in hyperexcitable hippocampal slices of kainate-treated rats. Eur. J. Neurosci. 8, 736–748 10.1111/j.1460-9568.1996.tb01259.x 9081625

[B38] Perin R. Berger T. K. Markram H. (2011). A synaptic organizing principle for cortical neuronal groups. Proc. Natl. Acad. Sci. U.S.A. 108, 5419–5424 10.1073/pnas.1016051108 21383177 PMC3069183

[B39] Santoro B. Baram T. Z. (2003). The multiple personalities of h-channels. Trends Neurosci. 26, 550–554 10.1016/j.tins.2003.08.003 14522148 PMC2924161

[B40] Sik A. Penttonen M. Ylinen A. Buzsaki G. (1995). Hippocampal CA1 Interneurons: an *in vivo* intracellular labeling study. J. Neurosci. 15, 6651–6665 7472426 10.1523/JNEUROSCI.15-10-06651.1995 PMC6577981

[B41] Silberberg G. Markram H. (2007). Disynaptic inhibition between neocortical pyramidal cells mediated by Martinotti cells. Neuron 53, 735–746 10.1016/j.neuron.2007.02.012 17329212

[B42] Singh A. Lesica N. A. (2010). Incremental mutual information: a new method for characterizing the strength and dynamics of connections in neuronal circuits. PLoS Comput. Biol. 6:e1001035 10.1371/journal.pcbi.1001035 21151578 PMC3000350

[B43] Tort A. BL. Rotstein H. G. Dugladze T. Gloveli T. Kopell N. J. (2007). On the formation of gamma-coherent cell assemblies by Oriens Lacunosum-Moleculare interneurons in the hippocampus. PNAS 104, 13490–13495 10.1073/pnas.0705708104 17679692 PMC1948921

[B44] Traub R. D. Jefferys J. G. Whittington M. A. (1999). Functionally relevant and functionally disruptive (epileptic) synchronized oscillations in brain slices. Adv. Neurol. 79, 709–724 10514857

[B45] Van Vreeswijk C. Abbott L. F. Ermentrout G. B. (1994). When inhibition not excitation synchronizes neural firing. J. Comput. Neurosci. 1, 313–321 10.1007/BF00961879 8792237

[B46] Varga V. Hangya B. Kránitz K. Ludányi A. Zemankovics R. Katona I. (2008). The presence of pacemaker HCN channels identifies theta rhythmic GABAergic neurons in the medial septum. J. Physiol. (Lond.) 586, 3893–3915 10.1113/jphysiol.2008.155242 18565991 PMC2538919

[B46a] Wang S. Chandrasekaran L. Fernandez F. R. White J. A. Canavier C. C. (2012). Short conduction delays cause inhibition rather than excitation to favor synchrony in hybrid neuronal networks of the entorhinal cortex. PLoS Comput. Biol. 8:e1002306 10.1371/journal.pcbi.1002306 22241969 PMC3252263

[B47] Wang X.-J. Buzsáki G. (1996). Gamma oscillation by synaptic inhibition in a hippocampal interneuronal network model. J. Neurosci. 16, 6402–6413 8815919 10.1523/JNEUROSCI.16-20-06402.1996 PMC6578902

[B48] Whittington M. A. Stanford I. M. Colling S. B. Jefferys J. G. Traub R. D. (1997). Spatiotemporal patterns of gamma frequency oscillations tetanically induced in the rat hippocampal slice. J. Physiol. (Lond.) 502 (Pt 3):591–607 10.1111/j.1469-7793.1997.591bj.x 9279811 PMC1159531

[B49] Williams J. H. Kauer J. A. (1997). Properties of carbachol-induced oscillatory activity in rat hippocampus. J. Neurophysiol. 78, 2631–2640 9356412 10.1152/jn.1997.78.5.2631

[B50] Wolfart J. Debay D. Le Masson G. Destexhe A. Bal T. (2005). Synaptic background activity controls spike transfer from thalamus to cortex. Nat. Neurosci. 8, 1760–1767 10.1038/nn1591 16261132

[B51] Zullo L. Chiappalone M. Martinoia S. Benfenati F. (2012). A “spike-based” grammar underlies directional modification in network connectivity: effect on bursting activity and implications for bio-hybrids systems. PLoS ONE 7:e49299 10.1371/journal.pone.0049299 23145147 PMC3493547

